# Merging Minds: The Conceptual and Ethical Impacts of Emerging Technologies for Collective Minds

**DOI:** 10.1007/s12152-023-09516-3

**Published:** 2023-03-28

**Authors:** David M. Lyreskog, Hazem Zohny, Julian Savulescu, Ilina Singh

**Affiliations:** 1 Department of Psychiatry, Warneford Hospital, University of Oxford, Warneford Ln, Oxford, OX3 7JX UK; 2 Oxford Uehiro Centre for Practical Ethics, University of Oxford, Oxford, UK; 3 Wellcome Centre for Ethics and Humanities, University of Oxford, Oxford, UK; 4 Centre for Biomedical Ethics, Yong Loo Lin School of Medicine, National University of Singapore, Singapore, Singapore; 5 Murdoch Children’s Research Institute, Melbourne, Australia; 6 University of Melbourne, Melbourne, Australia

**Keywords:** Brain-Computer Interfaces, Brain-Brain Interfaces, Collective Agency, Collective Responsibility, Swarm Intelligence, Hybrid Intelligence

## Abstract

A growing number of technologies are currently being developed to improve and distribute thinking and decision-making. Rapid progress in brain-to-brain interfacing and swarming technologies promises to transform how we think about collective and collaborative cognitive tasks across domains, ranging from research to entertainment, and from therapeutics to military applications. As these tools continue to improve, we are prompted to monitor how they may affect our society on a broader level, but also how they may reshape our fundamental understanding of agency, responsibility, and other key concepts of our moral landscape.

In this paper we take a closer look at this class of technologies – Technologies for Collective Minds – to see not only how their implementation may react with commonly held moral values, but also how they challenge our underlying concepts of what constitutes collective or individual agency. We argue that prominent contemporary frameworks for understanding collective agency and responsibility are insufficient in terms of accurately describing the relationships enabled by Technologies for Collective Minds, and that they therefore risk obstructing ethical analysis of the implementation of these technologies in society. We propose a more multidimensional approach to better understand this set of technologies, and to facilitate future research on the ethics of Technologies for Collective Minds.

## Introduction: The Problem of Collective Minds

Humans are social animals, and collaboration has enabled the survival and dominance of the human species. Consequently, there is an ever-growing literature seeking to pinpoint what exactly happens when we do things together – ranging from psychological accounts of communication and joint activity [13, 14, 51], to engineered strategies for improved collaboration [54], to philosophical and bioethical frameworks for how to conceptualize group agency and responsibility [1, 4, 47]. While there is some disagreement about the ontology and ethical relevance of groups and collective actions, we tend to hold that there is a relationship between individual and collective activities such that we could translate concepts for individuals – such as autonomy, identity, and responsibility – to groups and collectives. Others have emphasized the role and importance of relationships when it comes to understanding group agency and morality. While these accounts may well account for human morality so far, we may need to rethink this position in the light of emerging technologies for collective thinking, sensing, and decision-making – or, “Collective Minds”.

Since 2013, advances in technology continue to make this need evermore salient through the development of brain-to-brain interfaces (BBIs), which allow for the direct transfer of information between different brains [28, 30, 36, 38, 39, 45]. The potential for BBIs is vast, crossing the domains of medical treatment, human enhancement, warfare, and entertainment. For instance, they may enable us to boost sensory systems, such as by improving olfaction through linking one’s olfactory system to those of a dog [55], significantly speed up learning processes for sensorimotor tasks [39],and radically enhance decision making capacity by pooling information from vast numbers of connected brains working as part of a hive, mediated by algorithms [28].

Some authors have raised the usual ethical concerns with these prospects, such as safety, privacy, autonomy, research ethics, justice, and identity [8, 10, 26, 55]. These typically relate to questions about whether BBI users can adequately consent to participate (especially, for instance, if they suffer from a significant disability),whether sensitive or damaging information can be retrieved from participants’ brains; and whether users retain sufficient control over how their brain data is used. Possibilities such as hacking and a loss of control over action are also recurring concerns, as are broader questions related to who will have access to the technology, and on what basis.

However, not much attention has been directed toward whether our current accounts of collective intentions and actions are sufficiently well formulated to describe actions arising from BBIs and their potential successors. This, we will argue, is particularly relevant in prospective uses of BBIs which may involve inducing novel desires or intentions in the minds of recipients, and where it is unclear where those desires or intentions originate from. While some authors have recently highlighted the need to anticipate the future ethical impacts of these new interfaces, they tend to focus on the normative force of the endeavour itself, rather than on the conceptual reengineering which these technologies may entail, and the ethical impacts of those new conceptual constructs. For instance, Danaher & Petersen [10] recently argued for the desirability of a “hivemind society”, with Danaher strongly defending this view [9]. Although these contributions to the literature are valuable, there is a need for closer analysis of the conceptual and ethical impacts of technologies currently under development and in the pipeline.

In this paper, we first look at state of the art technology pertaining to collective thinking, sensing, and decision-making, its development potential, and how it may alter our understanding of how mental actions and decision-making works. We then investigate how collective and joint actions have typically been conceptualized. We argue that prominent and widely adopted accounts of such actions will struggle to relevantly describe actions performed by certain technologically-linked collectives, and that this hinders conceptual analysis and ethical assessment in the domain of what we will call “collective minds”. Finally, we highlight the ethical implications of the technologies in relation to the need for reconceptualization, and make some initial steps towards addressing them. We conclude that, given the state and direction of Technologies for Collective Minds (TCMs), there is an urgent need to anticipate not only the “usual suspects” in neuroethics (i.e. impact on autonomy, privacy, and so on), but to seriously reconsider the conceptual frameworks we use to describe phenomena arising from such technologies, so that conceptual analysis and ethical assessments can be made more relevant. We propose an approach to anticipating emerging issues with regard to the spectrum of TCMs emphasizing (a) dynamic reconceptualization of philosophical and ethical frameworks on pace with technological development, and (b) moving away from binary views on moral agency and responsibility, to better account for phenomena emerging from neuroengineering, and facilitate salient analyses of their social and ethical impacts.

## What is a collective mind?

We use the term “collective” to mean, roughly, two or more individuals acting together, though as we shall see, what counts as “acting together’” can be difficult to ascertain.

Tentatively, a “collective mind” can be defined as “a network of two or more individuals who are sensing, thinking, and/or making decisions together in real time”. Using this definition, one could argue that we have used tools for collective thinking for millennia. Scribbles and drawings have been found which date back over 73, 000 years [24], and “art” has been found which dates back to homo erectus, approximately 500 000 years ago [5]. Arguably, notes, drawings and art could be interpreted as powerful ways for minds to pass on knowledge and to work together across language barriers and generations [12, 33]. In the last few decades, the Internet and the communication platforms utilizing it have revolutionized our ability to perform collective actions beyond what was previously imaginable, offering vast amounts of information for free and enabling real-time collaboration on projects across the world. Yet, new and emerging technologies for collective thinking and action prompt us to ask if there’s yet another paradigm shift looming in the near future, in the realm of technologically supported collectivity.

For a number of years now, we have witnessed rapid developments in Brain-Computer Interfaces (BCIs). While these do not typically on their own reflect the sorts of technologies relevant to us here,^1^ they are a key starting block. BCIs are typically characterised by computer-based systems recording and analysing brain signals, and translating them into commands relayed to output devices to carry out desired actions [48].

With the rise of BCIs, another stream of technologies has gained traction which are less concerned with controlling external devices and more with direct access to other brains. By combining a BCI connected to the “sender” and a Computer-Brain Interface (CBI) connected to the “receiver”, a direct link between two or more brains can be established. Commonly referred to as, Brain-To-Brain Interfaces, or simply Brain-Brain Interfaces (BBIs), the computer component records and analyses neural activity from one brain and sends it to another, and (sometimes) vice versa. Multiple animal studies have been carried out over recent years, suggesting collaborative networks can improve task outcomes across a variety of domains [38, 44, 62]. BBIs have also be used in humans to solve collaborative cognitive tasks. In 2019, BrainNet was presented – a multi-person BBI for human collaboration [28]. A combination of EEG and transcranial magnetic stimulation (TMS) was used to simultaneously receive and send signals between the brains of the participants. Participants in the initial study collaborated to play a game of a digital Tetris-like game:

“Two of the three subjects are designated as ‘Senders’ whose brain signals are decoded using real-time EEG data analysis. The decoding process extracts each Sender’s decision about whether to rotate a block in a Tetris-like game before it is dropped to fill a line. The Senders’ decisions are transmitted via the Internet to the brain of a third subject, the ‘Receiver,’ who cannot see the game screen. The Senders’ decisions are delivered to the Receiver’s brain via magnetic stimulation of the occipital cortex. The Receiver integrates the information received from the two Senders and uses an EEG interface to make a decision about either turning the block or keeping it in the same orientation. A second round of the game provides an additional chance for the Senders to evaluate the Receiver’s decision and send feedback to the Receiver’s brain, and for the Receiver to rectify a possible incorrect decision made in the first round” [28].

In this manner, direct brain-to-brain communication could help with problem solving tasks in a variety of domains, including but not limited to: improved decision-making and coordination in military operations [2], communication with patients living with locked-in syndrome [26] – or simply to play games [28, 45].

Now, we can anticipate an emerging category of technology, namely Brain-Computer-Brain Interfaces (BCBIs) where the computer component functions not only as a mediator between humans – as in BBI – nor as a cognitive extension tool of a person, but as a “co-thinker” of sorts, organizing, optimizing, and potentially adding content to multi-person interfaces [58]. While applications for this class of technologies are currently lacking, the possibility is far from science fiction. In parallel with the engineering of brain interface technologies, there have been impressive developments in the field of mapping collective behaviours in animals, and in programming algorithms to mimic these behaviours to improve outcomes in collective tasks. Sometimes referred to as “swarming” or “swarm intelligence”, applied tools utilizing these algorithms have been shown to improve decision-making across a diverse range of domains in experimental studies, ranging from predicting outcomes of sports events to improving pneumonia diagnosis, and organizing policy priorities [41, 46, 59]. These developments are part of a broader line of research into “hybrid intelligence” – networks of humans and computers thinking together. It is plausible that swarm intelligence and other hybrid intelligence systems will be integrated into brain interfaces to improve decision-making mechanisms, giving rise to BCBIs. These networks will likely pose somewhat different conceptual and ethical problems which we are currently poorly equipped to deal with. More on this below.

For now, we can tentatively conceptualize TCMs as: “technologies which facilitate and/or enable networks of two or more individuals who are sensing, thinking, and/or making decisions jointly in real time”. TCMs may be constituted in numerous ways, which all have different ethical implications. Building on the terminology sketched by Nam and colleagues [36], we can conceptualize a scale based on *directness*, where technologies for collective thinking and acting may be more or less direct in nature, and a scale of *directionality*, which defines the direction of data flow. *Direct* interfaces involve directly stimulating the brain of a “receiver” to impart information, while *indirect* ones are those which rely on any other method than direct brain stimulation, such as speech or writing [36]. While there is some debate about the moral relevance of directness in brain interfaces, the common view is that direct (or highly direct) interference with neural activity is potentially more ethically problematic than indirect (or less direct) interference, usually due to concerns about free will and/or autonomy [15, 20, 21, 43]. Furthermore, it is important to note that directness and indirectness is not an either-or matter, but indeed a matter of scale – particularly on a systems level,the level of directness depends on the type of input and output (a sensory cue, for instance, may be taken to be less direct than an alteration of preference but is also affected by the quantity of highly direct connections within that system.^2^

The scale of *directionality* is defined by the direction(s) in which information is flowing, where *unidirectional* networks transfer information from a sender X to a receiver Y, and *multidirectional* networks where X, Y and potentially others are sending information to – and receiving information from – each other.^3^

It should be noted that directionality is not only a matter of network size, but that it also involves number of connections, data size and type, and levels of symmetry in the information flows. In a completely symmetric system, information flows equally between participants, i.e. data packages are sent and received in packages with corresponding data between all network participants. In an asymmetric system, conversely, forms of information may flow to or from its various participants to a higher or lesser extent, making the system epistemically and/or functionally unbalanced in terms of who sends, receives, and processes what (amounts and types of) data. The larger the system, the more complex the network’s directionality risks becoming in terms of data distribution.

**Fig. 1:**
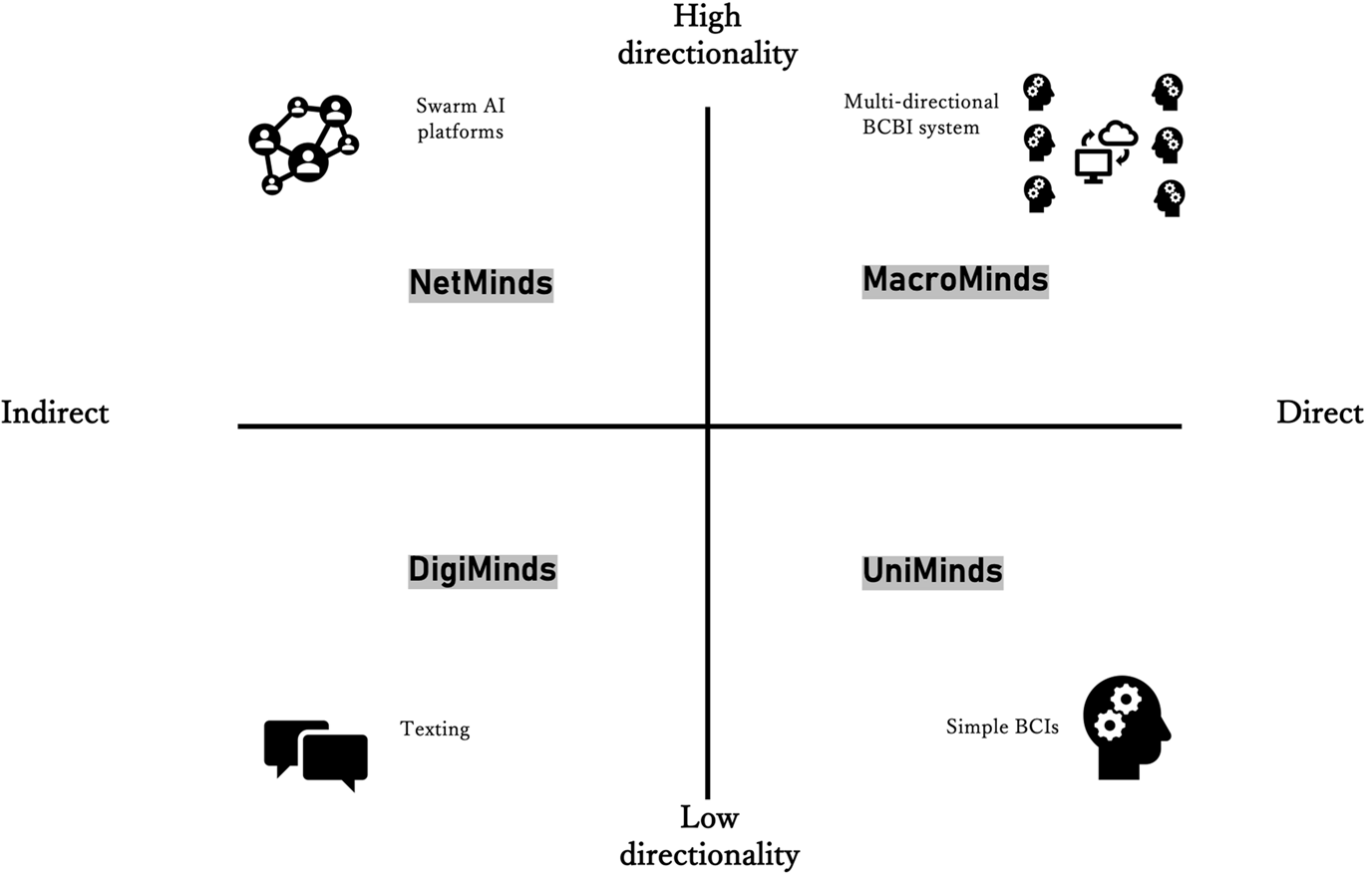
Visual conceptualization of Technologies for Collective Minds

Using the two scales of directness and directionality, we can visualize a spectrum across which TCMs may be more or less direct, and more or less multidirectional. We can then imagine at least four distinct forms of technology aiming to facilitate collective thought, sensation, and action (See Fig. 1).

1. DigiMinds

Minimally direct and minimally directed tools, which serve as communication platforms between users of the tool. These include many of the contemporary social and communication media platforms we use every day. While these technologies have their own important ethical landscapes, they are perhaps less interesting from the perspective of emerging, collective minds. Indeed, they arguably do not constitute collective minds at all, given our definition above.

2. UniMinds

Low-directional but highly direct brain-to-brain interfaces, where a (few) sender(s) communicates and manipulates the neuronal behaviour of a (few) receiver(s). Most of the current state-of-the-art BBIs fall within this category. UniMinds can be further divided into two sub-categories: (1) *weak* UniMinds, which are collaborative interfaces between two or more individuals, and (2) *strong* UniMinds which are intimate interfaces giving rise to a (new) joint entity.

3. NetMinds

Minimally direct, but highly directional technological tools can support vast networks of collective thinking by facilitating information transfer and deliberation. Prime examples of technologies which could enable NetMinds include “Swarm Intelligence” applications and other algorithmically powered software which seeks to collate and consolidate data from multiple users for seeking consensus in – and optimizing decision-making within – a given network.

4. MacroMinds

Maximally direct and maximally directed tools, which involve multiple participants connected through an interface which allows direct neuronal transmissions in all directions (i.e. as sender, computer, and receiver). This is the most underdeveloped category of technology of the four, and yet the ethically most challenging. Furthermore, as with UniMinds, we can conceive of two sub-categories of MacroMinds: (1) *weak* MacroMinds, which are collaborative interfaces between two or more individuals, and (2) *strong* MacroMinds which are intimate interfaces giving rise to a (new) joint entity.

Below we shall develop these categories further, looking at how they may be understood and how they may impact the ethics of TCMs. For now, with these four categories in place, we can begin to see how the emergence of technologies for collective thought, sensation, and action gives rise to a spectrum of more or less complex conceptual agent compounds. One may argue that we are currently at a stage of technological development which doesn’t warrant the term “collective mind” in any robust way, as contemporary tools are better described as means of communication than exhibits of genuine collective thought. That is, perhaps we are in an era of technologically *connected* minds rather than *collective* minds. Indeed, drawing a parallel to the terminology suggested above, we currently mostly use “DigiMinds” – minimally direct and minimally directional technologies – as means of communication. While this may be the case, we ought at the very least anticipate the impact of more intimately direct and multidirectional applications given the direction of current research efforts.

While there are important issues to address and resolve across the entire spectrum portrayed above – from Swarm Intelligence networks to BCI systems – one corner of the spectrum gives rise to particular concern; the more we move from indirect and unidirectional towards direct and multidirectional tools, the more muddled our sense of identity, agency, and responsibility seems to become. The problem with this is that widely adopted ways of thinking about joint and collective agency and responsibility do not accurately capture the decision-making processes leading up to actions carried out using certain TCMs. Consequentially, the difficulty of untangling and addressing ethical issues increases in parallel [10, 26, 55], 2015). To attend to this problem, we need to figure out how to conceptualize the emergence of collective minds and the implications of them. Let us first look at how we typically conceive of collective agency.

## On the Concept and Ethics of Collective Minds

A key concept for this domain, “agency” can denote a broad range of traits, capacities, and aspects of personhood. In discussing the potential impacts of TCMs, two aspects of agency are of particular interest. Firstly, the form(s) of agency commonly referred to as “moral agency” [27, 40], as contrasted with “causal agency” [49], agency as “rationality”, “control”, or combinations of these [29]. While the exact criteria for agency per se is an important and interesting field of research, for the purposes of this paper let us assume that moral agency can be held by any person who can be appropriately ascribed moral responsibility, blame, or praise – whatever we take the exact underlying criteria for that appropriateness to be. Furthermore, a key distinction commonly made is that between *individual* moral agency, and *collective* moral agency. Within philosophical, socio-psychological, and bioethical discourse there are numerous accounts seeking to describe and ascribe collective thinking, action, and responsibility across various domains: what does it mean for a corporation to act responsibly?,what is the nature of agency of a group of demonstrators?,can an idea be collective? Despite the richness of relevant literature, it is not clear that commonly accepted accounts of collective thinking, moral agency, and responsibility provide a framework that is conducive to ethically evaluating the implications of the TCMs described in the previous section. Commonly, such accounts point to that a collective action is only collective (and, by extension, there is a collective agent) if all members are aware that they are part of a particular collective, intend to act as that collective, and contribute to the action in question. On one of the most famous accounts of joint intentionality, Michael Bratman [4] argues that “we intend to J” (where “J” designates a joint action) if, and only if:

1. I intend that we J; and (b) you intend that we J.
2. I intend that we J in accordance with and because of 1a and 1b, and meshing subplans of 1a and 1b; you intend the same.
3. 1 and 2 are common knowledge.

John Searle [47], similarly, argued that persons participating in a joint action all must have intentions of the form “we intend to J”, in order for that action to be genuinely joint, and that “collective intentionality presupposes a background sense of the other as a candidate for cooperative agency; that is, it presupposes a sense of others as more than mere conscious agents, indeed as actual or potential members of a cooperative activity” ([47], p. 414).

Most contemporary accounts of joint action and agency build on more or less elaborate versions of this line of thinking – that joint action requires a “we-intention” or at the very least a “‘we-sensation” [1, 16, 18, 56]. What it means for a group of individuals to be considered as (and act as) an agent typically relies on the notion that (i) all members are aware that they are part of that collective and identify as such; (ii) all members intend to perform the action as that specific collective, and (iii) all members contribute to the realization of that action. But with TCMs, although it is plausible that some networks will allow or even require a we-intention or a sensation of jointness, it is equally plausible that other interfaces will not involve any such intentions or give rise to corresponding sensations. For instance, consider the technology used in the experiments by Ramakrishnan and colleagues [44], where monkeys were connected through a direct BBI to collaborate on a task which none of them could solve alone. Three monkeys (A, B, and C) controlled a marker which, if led to the right location (which changed after each successful marking) would give them all a treat. The marker was controlled via a brain interface, but the tricky part(!) was that each monkey could only control two axes: A could control axes X and Y,B could control axes Y and Z; C could control Z and X [44].

These monkeys did not intend to act together, as they were completely unaware of each other’s existence. Yet, it seems that they were solving these tasks as a collective rather than as separate entities. Likewise, for humans; while there is a distinct lack of studies on the phenomenology of BBIs, we can look to BCIs, where ethical as well as practical issues often stem from a distinct lack of integration, generating feelings of alienation. Conversely, where there is a certain level fluidness or “transparency” in user experience, usually due to users getting time to adjust to using the tool, said issues diminish [17, 23]. In cases of transparent utility, we rarely think that we are acting together with our computers – we simply act. So why would it be any different if we are hooked up to a BBI or BCBI network trying to, say, solve a complex task?

In this way, literature on collective and joint agency seems maladapted in the context of emerging TCMs: these technologies appear to allow us to act together through collaborative networks in highly integrated ways which seem to warrant collective agency, but our current models for describing (the constituents of) collective agency do not cover (all) such networks, but rather exclude them by definition. There are at least two reasons for this. The first, which has been covered to some extent above, has to do with the intent and/or sensation to act collectively. While our frameworks for collective action rely on these notions, TCM networks will not necessarily bring about such phenomena. Much like the monkeys acting to receive a reward, humans participating in a collective mind trying to solve a puzzle, give an accurate medical diagnosis, or predict financial markets, will not necessarily feel as if they’re doing something collaboratively. Nonetheless, their individual neural networks may all be closely interlinked to a point where it is not discernible who contributed with what neural processes, and which of these processes were sufficient or necessary for a task – or set of tasks – to be completed.

This brings us to the second reason, which has been less explored, namely that of opaque ownership of intentions, sensations, and (partial) actions. In trying to explain an action and the agency to which it is associated, current accounts do not easily lend themselves to the problem of identifying whether it makes sense to describe ideas or intentions developed within a collective mind as “one’s own”. In the case of, say, a large NetMind using some form of swarming algorithm to come to a decision using neural input from thousands of participants, it may be that nobody in that NetMind will identify as having made – or tried to make – that decision. In these cases, there will be a lack of intended collective action, as prescribed by accounts based on Bratman’s [4]. Yet, it seems prima facie true that the participants and/or the NetMind as a whole has made this decision.

This problem, of course, carries over into our conceptions of responsibility. These two domains – collective agency and collective responsibility – are deeply intertwined, but conceptually and pragmatically separate: one can be part of a joint action without being responsible for it, for instance by possessing low levels of agency or autonomy. Conversely, one can share a collective responsibility for the collective effects of parallel actions which are not connected through shared intentions. Blomberg and Hindriks [3] showcase this distinction between collective action and collective responsibility quite well, as they argue that agents with shared intentions are *more* blameworthy for morally problematic actions than agents who only act in parallel – although both sets of agents have *some* collective responsibility for their actions. Interestingly, they use an example including robots to highlight one of their points:

“[T]he involvement of others’ agency in bringing about a bad outcome does not itself decrease or dilute— nor increase or concentrate— an agent’s blameworthiness for bringing about that outcome. Consider a case where I intentionally bring about a bad outcome together with other agents. Now, replace those other agents with non- agential contributing causal factors (say, advanced robotic devices), but keep my intention and contribution fixed. There seems to be no reason why I would be any less or more blameworthy in the former case than in the latter just because some of the contributing causal factors happen to be other intentional agents rather than advanced robotic devices” ([3], p 146).

But it is far from clear that there is a *collective* responsibility at play at all in the robotics case, or whether there could be if the robots were to achieve agency through the interaction with human agents. It is also notable that Blomberg and Hindriks [3] base their work on Bratman’s reductionist account, and in doing so presume that collective responsibility can be exhaustively explained by the responsibility of the individuals constituting that collective. But this would remain a point of dispute in the context of TCMs, and in particular MacroMinds.

Levy and Giublini [19] have argued that “genuinely collective responsibility” can only be applied to *deeply collective* forms of joint action. This means two conditions must be satisfied. First, “for attribution of genuinely collective moral responsibility, the responsibility in question must not be reducible to a mere aggregation of individual responsibilities. […] An entity cannot be held morally responsible if *it* doesn’t have an identity and an intention or if *it* doesn’t perform the action for which it is held responsible.”

Second, “that a collective that has its own identity, its own intentions and performs its own action is appropriately held responsible in a way that accounts for its collective character, i.e. for the fact that the collective is constituted by a number of individuals. In other words, for its responsibility to be genuinely collective, rather than individual responsibility scaled up, this kind of responsibility must be attributed to the collective as a composite, and not an individual, entity: it must not be a form of individual responsibility that happens to apply to collectives.”(p.195).

Levy and Giublini make the case that *corporate* responsibility, for instance, is not a case of *collective* responsibility, arguing that “the responsibility in question is better understood as a type of individual responsibility scaled up and attributed to a peculiar type of agent, rather than as a form of genuinely collective responsibility.” [19], p 2018) It is unclear, however, whether TCMs could possibly constitute a basis for collective responsibility. I.e., should the individuals of a TCM network be held responsible for the actions of that network (a) as distinct individuals, (b) as constituents of one “macro-individual”, or (c) as a *genuine* collective?

Particularly in highly direct CMs, e.g. MacroMinds, it may be tempting to rely on single-brain concepts of responsibilization. One may ask what distinguishes a smaller neural network (single-brain) from what is essentially a larger neural network (multi-brain) in such a way that adoption of individualist concepts of autonomy, responsibility, privacy, etc., seem to be appropriate in former case but not in the latter. For instance, we can conceive of an approach to responsibility ascription which relies on a combination of the control condition for moral responsibility [50] and an understanding of being-in-control as one’s cognitive control network (CCN) being “in charge.”^4^ While this could be the case in some applications, it is not certain that any one CCN will be in charge of a given action performed by a MacroMind. For instance, it may be that (a) multiple CCNs are in charge of smaller sub-actions, where the (joint) goal of the total action is unclear or opaque,(b) CCNs are in charge of actions which are different or unrelated to the goal of the total action; (c) some CCNs are in charge of actions which de facto do not contribute to the joint action, albeit not for the lack of trying; (d) CCNs are unaware of an action being carried out at all (but perhaps aware that actions could potentially be carried out) and yet contribute to them in a significant way. The list goes on; there are a myriad of ways in which the tools we currently use to determine agency and responsibility in persons do not (at least not trivially) solve queries about the same in TCMs.

In this way, we can see how adopting contemporary ways of thinking about the concept of collective minds affects our ability to apply apt ethical frameworks in anticipating the relevant emerging technologies for collective sensation, thinking, and decision-making. We therefore need to rethink the concept of collective minds if we are to enable relevant ethical assessment of said technologies and their applications. It remains an open question, however, what exactly the ethical issues may be, and how they may or may not differ from those of other technologies currently in use. Establishing a review over ethical concerns raised in the literature on BCI technology, Coin, Mulder and Dublević [8] identify a number of areas which are frequently discussed:

Physical factors

- User safety

Psychological factors

- Humanity and personhood
- Autonomy

Social factors

- Stigma and normality
- Privacy and security
- Research ethics and informed consent
- Responsibility and regulation
- Justice^5^

While these areas of concern are specifically in the domain of BCIs, some of the ethical issues carry over to the domains of (other) TCMs. Indeed, Trimper, Wolpe and Rommelfanger [55] raise concerns about BBI technologies which risk blurring the line between “I” and “we”, particularly highlighting risks to privacy, agency, and identity, but also examining concerns about the possibility of BBIs being used as forms enhancement, and potential ethical issues in cross-species BBIs. Similarly, Hildt [25, 26] argues that the agency, responsibility, and identity of individuals comprising a BBI is at stake in numerous ways – in human–human interfaces, but also in networks including machine intelligences and/or animals. What these investigations into the ethics of brain interfaces have in common is that they apply widely used concepts of self, agency, and privacy to a new domain. Yet, little attention is given to the exploration of those concepts. If, as it seems from our analysis above, the underlying foundational concepts pertaining to self and collectivity are ill equipped to facilitate our understanding some emerging TCMs and their ethical implications, then we arguably need to first equip ourselves with more apt concepts before attempting an ethical analysis.

John Danaher and Steve Petersen [10] address this issue, as they ride out “in defence of the hivemind society”. Rather than a dystopian futuristic vision, they argue, the prospects of humanity moving towards a technology facilitated hivemind society may be quite bright. In doing so, they seek to define what a “hivemind” might be taken to be using the two parameters of unity in rationality (common and shared goal pursuit), and unity in phenomenology (shared sensation). Along these parameters, a society may be more or less “hivemind-ish”, depending on to what extent they display these two forms of unity.

Speculating about the ethical landscapes of societies with high levels of unity – that is, societies in which individuals intimately share goals, intentions, and phenomenal experiences – Danaher and Petersen [10] argue that a hivemind society may be desirable for a number of reasons, including that such a society would increase the goods of intimacy and goal achievement, and that it would free us from the chains of individualism:

“Individualism favours partiality, self-serving bias, and illusions of responsibility and virtue. It is because so many of us are trapped inside an individualistic bubble that we cannot act with true impartiality. [P]ursuing the hivemind ideal in both of its forms, would help us to shatter the illusion of independent selfhood and live a more altruistic and enlightened life in the sense that eliminating the distinctions between different agents, and seeing everyone as part of a single unified hivemind, would be to achieve a perfected form of impartial altruism” [10].

Although it is not clear that clear how exactly a collective (or hive-) mind would move us away from individualism and towards a more impartial morality,^6^ Danaher and Petersen here capture a crucial notion: it is not clear that a collective mind (or a “hivemind”) should be ethically assessed in the same way we assess individuals, or even other forms of collective structures (e.g. groups or corporations).

## Rethinking Collective Minds

The insight that our concepts of collective agency and responsibility must change to accommodate for certain sets of emerging technologies gives us a frame for how to define those very same technologies; we may narrow down the concept of “Collective Minds” to encapsulate (only) those technologies which, by design, more or less incapacitate our conceptual tools for analysing collective agency and responsibility:

A *Collective Mind* (a) is a network of two or more individuals who are sensing, thinking, and/or making decisions in real time, which (b) challenges models of collective action and decision-making, and (c) is constituted in such a way that no intent to collaborate or sensation of collaboration is necessary for a collective action to be taken.

In this way, the concept of “Collective Mind” is defined by the very problem it gives rise to, namely the challenge to common models for collective agency, and the inaptness of contemporary conceptual tools to help ethically evaluate the actions and designs of such minds. As emphasized earlier in this paper, some TCMs we have looked at may allow or give rise to we-sensations and -intentions – but not *necessarily*, and yet they may lead to events and actions which seem to us distinctly collective.

So how should we conceptualize the landscape of collective minds to help anticipate and assess ethical issues which may arise as TCMs are introduced across multiple domains? In order for this to be at all possible, we need to rethink our approach to identifying a collective mind, and distinguish between any relevant sub-categories within this domain.

One way of doing this is to build a framework based on the two scales presented in Sect. 2 of this paper: directness and directionality. As we could see (Fig. 1), a technology can be more or less *direct* in terms of recording and stimulating neuronal activity, and more or less *directed* in terms of to what extent participants of a network are senders, computers, and/or receivers of neuronal information. The further out on an axis an application is located, the more we have reason to raise concerns about the ethical aspects of that application; in particular applications which are both highly direct *and* highly directional. We identified four overlapping but distinct categories of technologies, to assist our thinking: Digiminds, UniMinds, NetMinds, and MacroMinds. In this section, we shall look a bit deeper at the similarities and differences between these categories, what the ethical relevance of the differences may be, and how these concepts can help us rethink our approach to assessing the impact of collective minds. First, however, we should add a couple of points.

First, beyond the two the two axes of directness and directionality, a third parameter may require consideration in anticipating the conceptual and ethical impacts of TCMs, namely the role of the non-human computer: artificial and ambient intelligence, algorithmic structures, and other technological capacities may give rise to additional conceptual and ethical concerns. While a computer ‘merely’ translating the signals between two brains may not raise any eyebrows, the addition of a complex algorithm for collating brain data and determining the best course of action, for instance, may give rise to concern. It is also worth noting that different types of connections – particularly in UniMinds and MacroMinds – are bound to yield different ethical issues: e.g. direct control over neuronal behaviour, motor information collection and processing, and mental content information and processing, will present separate ethically salient issues. More on this below, in connection to discussions on the four categories of TCMs.

Furthermore, as noted in Sect. 2 of this paper, there’s the issue of symmetry in multidirectional systems. While a flat network may yield fewer many conceptual or ethical concerns, the possibility of asymmetry echoes issues about epistemic justice in online environments [37, 53], and further obscures the nature of identity ascription: it may be that only a handful of participants of, say, one thousand network participants receive all data, while the remaining are only receiving some form of data, and sending a third form of data. The path to figuring out who is performing a specific cognitive task in such a scenario, and subsequently who is morally responsible for doing so, is going to be increasingly opaque the more complex the system is.^7^

Notable about all the four categories is that there are no requirements for we-sensations or we-intentions – the categories are defined by their technological capacities, not by their potential phenomenology. We have argued above that not all TCMs are likely to necessarily give rise to a we-sensation, or a we-intention, but that it nonetheless intuitively seems as if at least highly direct networks (e.g. UniMinds and MacroMinds) are capable of having intentions and carrying out actions which are collective in nature. Some TCMs may then give rise to we-sensations, while others may not. Yet, it is unclear whether that – the presence of a we-sensation – is of moral significance in this context. That is, all other things equal, are MacroMinds *with* we-sensations relevantly morally different in any way from MacroMinds *without* such sensations? While some have argued that this sort of phenomenal unity has a direct impact on the normativity of collective minds (Danaher, in Danaher & Nyholm [9, 10], the authors of this paper see the moral significance of that phenomenology as an open question. One could make an argument similar to that of Blomberg and Hindriks [3] (as quoted in Sect. 3 of this paper), that whether or not we *feel* as if we are bringing about an outcome together with other agents or by the use of non-agents is irrelevant to our de facto responsibility for contributing to bringing about that outcome. As a consequence, not only would it then be likely that many collective minds will not give rise to any we-intentions or -sensations – attributes which most prominent accounts of collective action and responsibility rest on – but the presence of such sensations and intentions would be ethically irrelevant. The authors of this paper refrain from taking any strong stance on this matter until further empirical evidence on the phenomenology of TCMs has been collected.^8^

Having said this, let us now have a look at the four distinct categories of TCMs which we will use to illustrate the conceptual reengineering of collective minds, and its ethical impacts.

### The concept and ethics of DigiMinds

We have defined DigiMinds as “minimally direct and minimally directed tools, which serve as communication platforms between users of the tool”. While many technologies of today fit this description, they are unlikely to qualify TCMs. The reason for this is mainly that they typically do not meet criterium (b) of Collective Minds, in that they would require a we-sensation for an action to be considered collective. As an example, we can imagine a group chat where we discuss how to best solve a given problem. While we collaborate on this task by thinking and making decisions to solve the problem, there is no space for us to use a group chat for such tasks without in some way sensing that we are doing it as a group and intending to collaborate in doing so.

That being said, there is nothing in the definition of DigiMinds which obviously rules out the emergence of technologies which may bypass we-sensations. As a hypothetical case, we can imagine an app which is meant to support decision-making through the assistance of an advanced artificial intelligence (AI). Let us further assume that this AI does not meet any standard criteria for (moral) agency – so it is regarded as a tool and not an agent – but only just below a threshold value for aforementioned agency. It is then conceivable that, while not being an agent in itself, the AI may gain agency by virtue of being supported by its human collaborator. While this possibility may seem outlandish prima facie, it is perhaps not such an alien idea if we accept the increasingly popular view that the autonomy and agency of an individual can be (and indeed needs to be) upheld and/or co-constituted through the support of other individuals [7, 11, 34, 35]. This typically involves a collaborative effort to bring persons above some baseline threshold of competence, so as to enable informed consent processes.^9^ There is no obvious reason why this shouldn’t apply to non-human individuals, such as AIs. In such a case, we might end up making a decision *together* with the AI, whilst not having a we-sensation at all, but rather the sensation of using an intricate tool to solve a problem or help make a good decision.

While it is in principle possible that a DigiMind could give rise to a Collective Mind in this way, there is little evidence that technological progress is anywhere near the level of sophisticated AI required for us to be able to “support it into agency”, as it were. Therefore, while technologies which are both minimally direct and minimally directed may come with their own sets of ethical issues, those issues are not going to be caused by the technologies giving rise to Collective Minds anytime soon. However, this phenomenon of agency-through-support may, as we shall see below, be more salient in other constellations.

### The concept and ethics of UniMinds

We have defined UniMinds as "low-directional^10^ but highly direct brain-to-brain interfaces, where a (few) sender(s) communicates and manipulates the neuronal behaviour of a (few) receiver(s)”. We further distinguished UniMinds between two sub-categories: (1) Weak UniMinds, which are collaborative interfaces between two or more individuals, and (2) Strong UniMinds, which are intimate interfaces giving rise to a (new) joint entity.

Weak UniMinds are only weak in the sense that the level of unity achieved does not warrant the emergence of a new entity. That does not mean that these interfaces cannot give rise to intricate and intimate collaboration and a strong sense of togetherness, although current BBIs (which surely must be considered as “Weak UniMinds”, if anything) may not give rise to any such sensations. Strong UniMinds, on the other hand, are defined by this feature of adding a new, emergent entity to the equation. While Strong and Weak UniMinds are similar in that they may or may not give rise to we-intentions or -sensations – and therefore bring about any ethical issues which may be typical for Collective Minds – the possible emergence of a merged and/or new entity is crucial to our thinking about the ethical landscapes of TCMs. Rather than looking at these forms of constellations as “genuinely collective” (in the terminology of Levy and Giubilini [19], it may be the case that we should conceptualize UniMinds as just that: *united* minds, merged into one network where the interface is in principle little different than an artificial brainstem, essentially connecting multiple (at least three) hemispheres so that they function as one joint organism. If this happens, we must ask ourselves how to apply our ethical frameworks: should we treat a Strong UniMind as *one* individual, hosted in separate biological bodies? If so, the possibility of reversing or exiting the unification of the minds will immediately come to the forefront of our inquiry. For instance, if two individuals unify their minds using a BBI and perform an action in that state, and then disconnect from that BBI, are those two individuals (a) collectively responsible for the action (b) individually responsible for the action by virtue of now being two separate individuals, or (c) individually responsible for the action by virtue of having acted as one (unified) agent? Are the two individuals equally responsible? Or perhaps there is no individual who is responsible anymore, as the responsible agent (the UniMind) has technically ceased to exist? While we cannot develop a thorough account for how to treat these cases here, it is clear that we will likely be wrestling with issues related to identity splicing of this sort in a not-too-distant future.

### The concept and ethics of NetMinds

We have defined NetMinds as “minimally direct, but highly directional technological tools that can support vast networks of collective thinking by facilitating information transfer and deliberation”.

Prime examples of technologies which could enable NetMinds include “Swarm Intelligence” applications and other algorithmically powered software which seeks to collate and consolidate data from multiple users for seeking consensus in – and optimizing decision-making within – a given network. While these minimally direct technologies may not tickle our imagination as much as BBIs and other highly direct tools, they nonetheless stand to pose salient ethical challenges. Along with the fact that these technologies do not necessarily give rise to any we-sensations or -intentions, there are at least two aspects which make them tricky from an analysis and assessment point of view.

First, we need to consider the scale on which technologies for distributed deliberation and decision-making may operate. Much like the insect colonies and flocks of birds which many of these technologies are modelled on, there is no reason why hundreds of even thousands of individuals cannot participate in tasks in real-time, only focusing on a small part on their own, but on a large scale contributing to complex tasks and decision-making processes. What’s interesting is that these tasks probably can only be carried out because of this inherent aggregate intelligence design: it will plausibly be incomprehensible to any one individual participating in such a network why or how a certain goal was pursued by the collective, or why a given decision was made. Again, this challenges how we think about collective actions, as the cognitive processes underlying any actions taken by a NetMind would in principle be unfathomable to any one individual. This raises the question whether we can be responsible for participating in actions which we are in principle unable to fathom.

Second, there is the question of the role of the computer. For a NetMind to work as such, and to not collapse into a chaotic chatroom, there needs to be a computer which organizes the input and calibrates any feedback loops to optimize deliberation and decision-making – let’s call it an Organizer. The Organizer could be anything from an algorithm to an advanced general AI, as long as it serves its purpose of organizing information so that an optimal result is reached (or at the very least pursued). But the better the organizer is at its task, and the larger the pool of participants, the more difficult it is going to be to derive *why* certain decisions are made. The “black box problem” is commonly discussed in AI ethics, and highlights the issue that while an AI or an algorithm may lead to good (better-than-human) outcomes in diagnosis, predictions, and analyses, there is no way of telling how it came to do so, or why it chose a certain path. This problem may inflate in the context of NetMinds, where it is not clear how the preferences, thoughts, or estimates produced by a hundred or a thousand individuals in the NetMinds are weighed, or how a conclusion is reached. In these cases, not only would participants in a NetMind not know what decision is going to be made, but it may be completely opaque to them *how* that decision may be made.

Related to this last point, there is also the issue of the Organizer as a potential co-thinker and de facto agent. However, this point is more salient to the concept of MacroMinds, and we shall therefore discuss this more in detail below.

### The concept and ethics of MacroMinds

MacroMinds are maximally direct and maximally directed tools, which involve multiple participants connected through an interface which allows direct neuronal transmissions in all directions (i.e. as sender, computer, and receiver). Furthermore, as with UniMinds, we can conceive of two sub-categories of MacroMinds: (1) Weak MacroMinds, which are collaborative interfaces between two or more individuals, and (2) Strong MacroMinds which are interfaces giving rise to a (new) joint entity.

Arguably the most conceptually and ethically challenging set of TCM constellations, MacroMinds would require a network constituted by a large number of individuals, and (at least one) artificial intelligence tasked with organizing brain data, and potentially additional data external to human input, and reaching a decision for the MacroMind as a whole. As such, any problems identified for NetMinds and/or UniMinds are likely to be at play also for MacroMinds. While both Weak and Strong MacroMinds may or may not give rise to we-sensations or -intentions, and will therefore share a number of ethical challenges, for the sake of clarity, let us investigate them separately.

First, Weak MacroMinds do not warrant the ascription of a new (additional) entity, but will still disrupt our sense of identity and agency of a Collective Mind. For instance, while the potential agency of an Organizer is already an issue in less direct NetMinds, in Weak MacroMinds this issue appears quite salient. The collective mental capacities of multiple individuals are likely to not only be aided by the Organizer, but to aid it in return. In doing so, we may accidentally support the Organizer into agency, as it were. Much like if a person may struggle to make an informed and competent decision with regard to a complex medical procedure if she is not trained or experienced in doing so, she may be supported by medical professionals and persons close to her to gain a higher level of agency and to make a good decision, we can imagine that a non-agent Organizer may be supported into some form of agency if intimately and collaboratively connected to multiple (other) agents.

Strong MacroMinds, by definition, inherit the potential hazards of entity emergence as described in relation to UniMinds: how should we conceive of, and treat, MacroMinds which generate a new entity which is not necessarily permanent, but contingent on the continued connectedness of its constituents? Along with the difficulty of ascribing responsibility to these sorts of entities which we identified in the section on UniMinds, an additional difficulty more salient to MacroMinds is that of determining the origin of intuitions, thoughts, and decisions. It doesn’t appear to make sense to ask “who in this Mind decided X?” It appears more feasible, at least prima facie, to treat the MacroMind as one entity which has multiple constituents, and those constituents happen to be (potentially, partially) people. But we are then faced with the challenge of conceptually containing the identity of a MacroMind: under what conditions does a MacroMinds keep its identity over time?; how many (and which) constituents can be removed, added, or changed whilst maintaining the identity of a MacroMind? At its core, this is essentially an identity problem which we humans have wrestled with for hundreds, if not thousands of years, and was already described by Plutarch around the beginning of the second century AD in his stories of Theseus and his ship:

“The ship wherein Theseus and the youth of Athens returned had thirty oars, and was preserved by the Athenians down even to the time of Demetrius Phalereus, for they took away the old planks as they decayed, putting in new and stronger timber in their place, insomuch that this ship became a standing example among the philosophers, for the logical question of things that grow; one side holding that the ship remained the same, and the other contending that it was not the same” (Plutarch (retrieved 2022))

In the case of MacroMinds, and indeed other forms of networks formed through the use of TCMs, we may similarly ask when we should hold such a mind to be one and the same, and when to not do so, as constituent individuals may come and go throughout the lifetime of a MacroMind. While this is a problem in terms of defining the identity of a specific MacroMinds, it doesn’t necessarily translate to responsibility at the individual level. Much like if a constituent of my brain (e.g. a neural transplant) was not part of my brain at the time of a given action, we could still hold *me* responsible while at the same time agree that that part of my brain does not carry any part in that responsibility, individuals may not be responsible for the actions of a MacroMind they are part of, if they were not part of that MacroMind at the time the decision was made to perform that action. So far so good, but it is yet not clear when individuals *are* responsible, and whether it makes sense to hold them responsible as individuals, or indeed as constituents of one larger entity. One way to address this is to anchor the responsibility in the possibility of reversibility, i.e.: if we cannot untangle a given MacroMind, we have no choice but to treat it as one (macro-)individual. Whatever the duties, rights, and responsibilities such an individual may have, will in that case need to be determined in some way. If we, on the other hand, can (in principle) dismantle that MacroMind so that separate individuals re-emerge, we may hold them individually accountable as part of a collective or group. Perhaps we could build a framework for responsibility around the intentions and the foreseeability of any given action taken by the MacroMind: if they (the individuals) shared an intention to have the MacroMind cause an impact X, then they are responsible for X; If they do not intend but can *foresee* the causing of X, they are also responsible for X, but; they are not responsible for X if they cannot foresee that the MacroMind will have the impact X, or if they cannot foresee that they will become part of that specific MacroMind at all.

The analyses of these four archetypes of collective minds should not by any means be taken to be exhaustive of the types of constellations and ethical issues we’re bound to see emerge over the coming decades. They could, however, play an important role in helping us move away the conceptual binarity – individual or *collective* – dominating contemporary thinking about moral agency and responsibility, and towards a multidimensional approach which allows more agile and salient conceptual and ethical analysis.

## A new field

In this paper, we have argued that new and emerging TCMs challenge commonly held views on collective and joint actions in such a way that our conceptual and ethical frameworks appear unsuitable this domain. This inadequacy hinders both conceptual analysis and ethical assessment, and we are therefore in urgent need of a conceptual overhaul which facilitates rather than obstructs ethical assessment. In this paper, we have but taken the first steps to bring about this overhaul: while our four categories – DigiMinds, UniMinds, NetMinds and MacroMinds – can help us think about the dimensions of Collective Minds and their ethical implications, it remains an open question how we should treat TCMs, and which aspects of them are most ethically salient, as this will depend on a number of parameters, including (A) the technological specifications of any TCM, (B) the domain in which said TCM is deployed, (military, medicine, research, entertainment, etc.) and (C) reversibility (i.e. whether joining a given Collective Mind is permanent, or risk leaving significant permanent impacts). It is also worth recalling that these four categories, while based on technological capacities, are only conceptual tools to help navigate the ethical landscapes of Collective Minds. What we are likely to see in the coming years is the emergence of TCMs which do not easily lend themselves to be clearly boxed into any of these four categories, under descriptions such as “Cloudminds”, “Mindplexes”, or “Decentralized Selves” [22, 31, 32, 52, 61].

In anticipating and assessing the ethical impacts of Collective Minds, we propose that we move beyond binary approaches to thinking about agency and responsibility (i.e. that they are either individual or collective), and that frameworks focus attention instead on the specifics of ABCs as stated above. Furthermore, we stress the need to fluently and continuously refine conceptual tools to encompass those specifics, to adapt our ethical frameworks with equal agility.

## Data Availability

No data was collected in this study.

## Footnotes

1 Except perhaps in cases where the Computer in the BCI constellation is sophisticated enough to constitute an artificial mind.

2 There’s also something to be said about the *type* of connections which are at play. As one reviewer of this paper pointed out, different types of connections (control connections, information processing connections, etc.) can be made between system constituents – be they brains or computers. It remains unclear whether one type of connections should be considered *more* direct than another, but they are prima facie relevant to ethical analysis. More on this is Sect. 4.

3 It should be noted that Nam et al. [36] treat directness and directionality (“directedness”, in their terminology) as a matter of sharp distinction, not as scales, generating 4 distinct categories (direct/indirect, unidirectional/bidirectional, and their possible combinations). However, it is not clear that interfaces will always fall so clearly within these categories. Their definition of the term “directedness” leaves it open to ask what qualifies as “neuromodulation” on their account, and perhaps not all participants of an interface use the same tools to link up. Similarly, the directionality of an interface is a matter of degree in cases where participants’ control is asymmetrical. For instance, while X is using a unidirectional connection, Y and Z might be using bidirectional connections. In this way, interfaces may support networks which are direct and bidirectional to varying degrees.

4 We would like to thank an anonymous reviewer for raising this suggestion.

5 As one reviewer pointed out, these categories are not exclusive to their respective category, e.g. “Privacy” is also a psychological factor. Nor is the list exhaustive.

6 Indeed, as one reviewer pointed out, collective minds could also be individualistic and partial.

7 This challenge may apply to large, complex organisation like governments or corporations to some extent, too, but even in such cases there remains a possibility of tracing outcomes to individual wrong-doing. This may prove more difficult in complex TCM systems, where no particular individual can be identified as contributing to a bad outcome.

8 A reviewer noted that one area of research that may be instructive to this debate relates to craniopagus conjoined twins, whose brains are joined at the thalamus. For instance, Cochrane [6] argues for the possibility of shared consciousness among these twins. Further work could consider whether, and to what extent these may be analogous to minds arising from certain types of TCMs.

9 What exactly the criteria for those thresholds (i.e. thresholds of competence) are remains debatable, and will naturally differ between domains and contexts. Nonetheless, there seems to be a growing consensus that there *are* thresholds, and that persons can be supported into getting across them in decision-making scenarios.

10 Low-directional, we take it, would typically mean uni- or bi-directional networks between a small number of individuals.
